# Genotoxic and mutagenic properties of Ni and NiO nanoparticles investigated by comet assay, γ‐H2AX staining, *Hprt* mutation assay and ToxTracker reporter cell lines

**DOI:** 10.1002/em.22163

**Published:** 2017-12-15

**Authors:** Emma Åkerlund, Francesca Cappellini, Sebastiano Di Bucchianico, Shafiqul Islam, Sara Skoglund, Remco Derr, Inger Odnevall Wallinder, Giel Hendriks, Hanna L. Karlsson, G. Johnson

**Affiliations:** ^1^ Unit of Biochemical Toxicology, Institute of Environmental Medicine, Karolinska Institutet Stockholm 171 77 Sweden; ^2^ Division of Surface and Corrosion Science, School of Chemical Science and Engineering KTH Royal Institute of Technology Stockholm Sweden; ^3^ Toxys, Robert Boyleweg 4, 2333 CG Leiden the Netherlands

**Keywords:** genotoxicity, lung cells, nanomaterials, nickel, reporter cell lines

## Abstract

Nickel (Ni) compounds are classified as carcinogenic to humans but the underlying mechanisms are still poorly understood. Furthermore, effects related to nanoparticles (NPs) of Ni have not been fully elucidated. The aim of this study was to investigate genotoxicity and mutagenicity of Ni and NiO NPs and compare the effect to soluble Ni from NiCl_2_. We employed different models; i.e., exposure of (1) human bronchial epithelial cells (HBEC) followed by DNA strand break analysis (comet assay and γ‐H2AX staining); (2) six different mouse embryonic stem (mES) reporter cell lines (ToxTracker) that are constructed to exhibit fluorescence upon the induction of various pathways of relevance for (geno)toxicity and cancer; and (3) mES cells followed by mutagenicity testing (*Hprt* assay). The results showed increased DNA strand breaks (comet assay) for the NiO NPs and at higher doses also for the Ni NPs whereas no effects were observed for Ni ions/complexes from NiCl_2_. By employing the reporter cell lines, oxidative stress was observed as the main toxic mechanism and protein unfolding occurred at cytotoxic doses for all three Ni‐containing materials. Oxidative stress was also detected in the HBEC cells following NP‐exposure. None of these materials induced the reporter related to direct DNA damage and stalled replication forks. A small but statistically significant increase in *Hprt* mutations was observed for NiO but only at one dose. We conclude that Ni and NiO NPs show more pronounced (geno)toxic effects compared to Ni ions/complexes, indicating more serious health concerns. Environ. Mol. Mutagen. 59:211–222, 2018. © 2017 The Authors Environmental and Molecular Mutagenesis published by Wiley Periodicals, Inc. on behalf of Environmental Mutagen Society

## INTRODUCTION

Nickel (Ni) has been in widespread commercial use for the last 100 years due to important properties allowing, e.g., the production of stainless steel. Ni production and use has led to exposure of several millions of workers to Ni‐containing dust [IARC, 1990] and occupational exposure is associated with various pathological effects including skin allergies [Schram et al., 2010]. In addition, exposure to nickel‐containing particles has been shown to increase the risk for lung cancer in humans as well as in experimental animals [IARC, 2012; Oller et al., 2014]. The studies have in general indicated different potency depending on the solubility of the Ni containing particles [Goodman et al., 2011]. IARC (the International Agency for Research on Cancer) has classified Ni metal particles in Group 2B, i.e., possibly carcinogenic to humans [IARC, 1990], whereas “nickel compounds” have been classified in Group 1 (carcinogenic to humans) [IARC, 2012]. In addition to unintentionally formed Ni‐dust at occupational settings, Ni nanoparticles (NPs) are now also manufactured. Ni NPs harbor some unique and desirable characteristics that are only present at nanoscale, including a high level of surface energy, high magnetism, low melting point, high surface area, and low burning point, and are therefore used in different applications [Magaye and Zhao, 2012]. This intentional production of Ni NPs may lead to an increased risk of human exposure.

Despite the classification of nickel compounds as carcinogenic, the underlying mechanisms that may result in lung cancer are still poorly understood. Previous studies have suggested both direct DNA damage in the form of DNA strand breaks [M'Bemba‐Meka et al., 2005; Latvala et al., 2016] and indirect genotoxic effects due to inhibition of DNA repair [Schwerdtle et al., 2002; IARC, 2012]. Oxidative stress has been suggested to be an important underlying mechanism and increased levels of 8‐oxo‐2‐deoxyguanosine have, for example, been observed in the lung of mice exposed to different nickel compounds [Kawanishi et al., 2001]. In addition, gene silencing due to epigenetic effects has been suggested to be an important mechanism [Costa et al., 2005]. Overall, there is still a discussion regarding to what extent genotoxicity contributes to nickel‐induced carcinogenesis, and the number of studies investigating genotoxicity of Ni and NiO NPs is still limited.

The aim of this study was to investigate genotoxicity of Ni and NiO NPs compared to Ni ions/complexes from the soluble salt NiCl_2_. Different models and methods were employed with the intention to obtain a better overall picture of genotoxicity and its underlying mechanisms. These models included; (1) exposure of human bronchial epithelial cells (HBEC) followed by DNA damage analysis using the comet assay and γ‐H2AX (phosphorylated H2A histone family, member X) staining, (2) exposure of six different mouse embryonic stem (mES) reporter cell lines that are constructed to exhibit fluorescence upon the induction of various pathways of relevance for (geno)toxicity and cancer [Hendriks et al., 2016], and (3) exposure of mES cells (IB10) for mutagenicity tests by using the *Hprt* (hypoxanthine phosphoribosyl transferase) mutation assay according to OECD guideline (OECD 476).

The HBEC cells were used due to the fact that lung cells constitute a relevant model for investigating genotoxicity following inhalation. These cells (HBEC3‐kt) are normal human bronchial epithelial cells that have been immortalized by transfection with a retroviral construct containing cyclin‐dependent kinase (Cdk) 4 and human telomerase reverse transcriptase (hTERT). The cells do not form colonies in soft agar and they do not form tumors in mice, hence they are considered to display a non‐cancerous phenotype and are used as an *in vitro* model to mimic “normal lung cells” [Ramirez et al., 2004]. For mutations, the *Hprt* mutation assay was used since this is an OECD accepted method and furthermore since the more commonly used Ames test is not recommended for NPs due to limited uptake [Doak et al., 2012].

Besides these more traditional assays we employed six different green fluorescent protein (GFP)‐based reporter cell lines (called ToxTracker) to obtain further mechanistic insight. These reporter cells are based on mouse embryonic stem (mES) cells, which are genetically stable, proficient in all cellular DNA repair pathways and have a high rate of cell proliferation, which makes them sensitive to DNA damage [Giachino et al., 2013]. The assay procedure is very efficient; the reporter cells are exposed to the NPs in 96‐well plates and the fluorescence in live cells is examined by flow cytometry after 24 h. Two of the constructed reporter cell lines [Hendriks et al., 2016] are triggered by oxidative stress as a result of increased antioxidant signaling (*Srxn1* and *Blvrb* reporters).Two other reporter cell lines indicate DNA damage as a result of induction of signaling pathways for replication stress (*Bscl2* reporter) or to NFκB signaling (*Rtkn* reporter). These reporters are e.g., activated by genotoxic substances such as doxorubicin [Hendriks et al., 2016]. The remaining two cell lines indicate general p53‐dependent cellular stress (*Btg2* reporter) or protein unfolding (*Ddit3* reporter). The use of these reporter assays provides a more high‐throughput alternative compared with many other assays [Nelson et al., 2016]. We have previously elucidated the applicability of three of these reporters for NPs [Karlsson et al., 2014].

## MATERIAL AND METHODS

### Cell Lines

HBEC3‐kt cells, originally from ATCC, were kindly provided by Dr. Zienolddiny, Statens arbeidsmiljøinstitutt (STAMI), Norway. These cells were cultured at serum free conditions in 50% RPMI (Roswell Park Memorial Institute) medium, (Sigma Aldrich, St. Louis, MO, USA), supplemented with 1% L‐glutamine (Gibco, Thermo Fisher Scientific Inc., Waltham, MA, USA) and 1% PEST (penicillin‐streptomycin, Gibco), and 50% LHC‐9 (Laboratory of Human Carcinogenesis‐9) medium (Gibco) supplemented with 1% PEST. The cells were cultured in T75 flasks pre‐coated with 0.01% collagen (Type I, PureCol® from Advanced BioMatrix) and were split every 2–3 days. Culturing of the ToxTracker mES cells was performed as described previously [Hendriks et al, 2012]. The mES cells were maintained on 0.1% gelatin‐coated plates in the presence of irradiated mouse embryonic fibroblasts as feeder cells in KnockOut DMEM (Dulbecco Modified Eagle Medium, Gibco) containing 10% FBS (fetal bovine serum), 2 mM GlutaMAX, 1mM sodium pyruvate, 100 μM β‐mercaptoethanol (all from Gibco), and leukemia inhibitory factor (LIF, home‐made). KnockOut DMEM is a basal medium optimized for growth of undifferentiated embryonic and induced pluripotent stem cells. The cells were seeded 24 h prior to exposure on gelatin‐coated plates using buffalo rat liver cell (BRL)‐conditioned mES cell medium. V79‐4 cells (Chinese hamster lung cells), purchased from Sigma‐Aldrich, were cultured in DMEM (Sigma) supplemented with 1% PEST (penicillin‐streptomycin) and 10% FBS (fetal bovine serum), qualified US origin (Gibco). The cells were split every 2–3 days.

### Nickel Materials and Exposure

Nano‐sized Ni metal particle powder (<100 nm diameter, purity >99%, Cat#: 577995, 93397KJ), nano‐sized NiO particle powder, (<50 nm diameter, >99.8% purity, Cat# 637130, 17198PJ) and soluble Ni chloride (NiCl_2_·6H_2_O referred to as NiCl_2_) powder (Cat# N5756, 37H3494) were purchased from Sigma‐Aldrich. The powders were weighed and suspended in corresponding cell media at a stock concentration of 1 mg/mL. The particle suspensions were vortexed and sonicated in a water bath for 15 min at room temperature (RT). The particle suspensions were then diluted to100 μg/mL, and the appropriate volume for each exposure condition was added directly to the cells in the cell media. The cells were exposed to corresponding mass concentration of Ni, i.e., the mass of Ni in the case of NiO and NiCl_2._ Fresh suspensions were prepared for every experiment in the appropriate cell media.

Size and morphology of the same batch of Ni and NiO NPs, as used in this study, have been studied previously using TEM (transmission electron microscopy) [Kain et al., 2012; Latvala et al., 2016]. These analyses showed that the Ni NPs were of varying sizes, generally less than 100 nm, whereas the NiO NPs were less than 50 nm. The larger fraction of smaller‐sized NiO NPs is indicated by their higher specific surface area (determined by the Brunauer‐Emmet‐Teller method). Release of Ni from the same batches of NPs in serum‐containing DMEM (similar as the media used for culturing V79‐4 cells and the ToxTracker reporter cells in this study) has also previously been reported [Latvala et al., 2016]. A summary of these results is shown in Table 1.

**Table 1:** Summary of NP Characteristics; Particle Size, BET‐Area and Ni‐Release in Serum Containing DMEM as Well as Serum Free RPMI/LHC‐9 Medium

NP	BET‐area^a^ (m^2^/g)	Primary size^b^ (nm)	% Ni release in solution in DMEM (serum‐containing)^c^	% Ni release in solution in RPMI/LHC‐9 medium (serum free)
Ni	6.41	<100	∼2%	∼2%
NiO	102	<50	∼3%	∼6%

a Data reported in Latvala et al. (2016).

b TEM images shown in Latvala et al. (2016) and Kain et al. (2012).

c Data reported in Latvala et al. (2016) following 24 h exposure.

## ICP‐MS

The amount of Ni released in HBEC cell culture medium (RPMI/LHC‐9) was evaluated by inductively coupled plasma mass spectrometry (ICP‐MS). Ni and NiO NPs were dispersed (50 µg/mL, corresponding to total Ni) in cell culture medium and incubated at 37°C for 3 and 18 h. NPs were then separated from the released Ni fraction by using centrifugation at 13 400 rpm (5 min). Next, 600 µL of the supernatant was digested using 900 µL of 67% HNO_3_ for at least 24 h. The digested supernatant was then further diluted in 1% HNO_3_ (final dilution 20). ^58^ Ni and ^60^ Ni isotopes were quantified and run on KED mode on the ICP‐MS (iCAP Q; Thermo Scientific, Waltham, MA, USA). Calibration standards at 1, 5, 10, 50, 100, and 500 µg/L of Ni were prepared from a 1000 mg/L stock in 1% HNO_3_. As an internal standard all samples were spiked with 4.99 µg/L indium (999 ± 5 µg/mL; Lot: F2‐IN01094, Spectrascan, Teknolab AS, Norway) with a range of recovery between 100% and 108%. Three independent experiments were analyzed for the different time points. The mean values of isotopes were used and results were expressed as % of Ni release from the initial concentration.

## PCCS

Size measurements of the NPs were performed by means of photon cross‐correlation spectroscopy (PCCS). Ni and NiO NP suspensions were prepared as described above and diluted to final concentrations of 10 μg/mL in the medium used for the HBEC cells (RPMI/LHC‐9) and the ToxTracker reporter cells (knockout DMEM medium), respectively. The particle suspensions were then measured at the time points 0 h, 1 h and 24 h at 37°C with a Nanophox instrument (Sympatec). Standard latex samples (20 ± 2 nm and 100 ± 5 nm) (Sympatec) and blank samples were analyzed prior to the measurements to ensure a high accuracy of the measurements. Each sample was measured three times and the data from each of the separate measurements were combined to produce the final graph by using the software provided by Sympatec (WINDOX 5).

### Alamar Blue Assay

HBEC cells were exposed to Ni, NiO NPs and NiCl_2_ (5, 10, 25, and 50 µg Ni/mL; corresponding to 1.56, 3.13, 7.81, and 15.63 μg Ni/cm^2^) for 24 h in 96‐well plates, final volume 100 μL. Cell culture medium was used as a negative control and Triton X‐100 1% (Sigma‐Aldrich) was used as a positive control. After exposure, the suspensions were removed and the cells were incubated with 10% Alamar Blue (Invitrogen, Carlsbad, CA USA) for 2 h at 37°C. A microplate reader (Tecan, Infinite F 200, Software: Magellan 7.2, Austria) was used to read the fluorescence at 560 nm excitation and 590 nm emission. Background values (10% Alamar blue in cell culture medium) were subtracted from each well and the average fluorescent intensity of the triplicates was calculated. Three independent experiments were performed.

### Comet Assay

The alkaline version of the comet assay was used to measure DNA strand breaks. FPG (Formamidopyrimidine DNA glycosylase, kindly provided by Prof. A. R. Collins, Dep. Nutrition, School of Medicine, University of Oslo, Norway), which mainly detects purine oxidation products, was added to the assay for evaluation of oxidatively damaged DNA (FPG sites).

HBEC cells were exposed to Ni NP, NiO NP and NiCl_2_ for 24 h at doses of 5, 10, and 25 μg Ni/mL (corresponding to 1.58, 3.16, and 7.89 μg Ni/cm^2^), in 24‐well plates, final volume 600 μL. The cells were washed with PBS (Gibco) and harvested with trypsin (Gibco) and then processed for the comet assay. The mini‐gel comet assay as previously described [Di Bucchianico et al., 2017] was used and cells exposed to hydrogen peroxide (0.05 M) for 5 min on ice were used as a positive control. In short, following exposure, cells were embedded in 1.1% agarose mini‐gels on pre‐coated (0.5% agarose) microscope slides. The mini‐gels were allowed to solidify on a cold tray for a few min and the slides were then placed in lysis buffer, pH 10 (1% Triton X‐100, 2.5 M NaCl, 10 mM Tris, 0.1 M EDTA, Sigma‐Aldrich) for 1 h on ice at dark conditions. The slides were washed 3 times in enzyme buffer (0.1 M KCl, 0.5 mM EDTA, 40 mM HEPES, 0.2 mg/mL BSA, Sigma‐Aldrich). The FPG‐enzyme was diluted 1:1000 in enzyme buffer and 30 μL was added to each mini‐gel. 30 μL enzyme buffer was added to the control mini‐gels. The slides with the mini‐gels were incubated in a humidity chamber at 37°C for 30 min. The slides were then incubated in alkaline solution (0.3 M NaOH, 1 mM EDTA, Sigma‐Aldrich) for 40 min at dark conditions in the presence of cooling blocks, to unwind the DNA. Electrophoresis (29V, 1.15 V/cm electrophoresis platform) was performed during 30 min in an electrophoresis tank. The slides were neutralized in 0.4 M Tris for 5 min (2 times) and in water for another 5 min. Fixation was performed in methanol for 5 min. The slides were stained in a 1:10,000 dilution of Sybr Green (Invitrogen) in TAE buffer for 15 min and scored with a fluorescence microscope (LeicaDMLB, Houston,TX) using the Comet assay IV software (Perceptive Instruments, Suffolk, UK). In each experiment 50 comets in duplicates on two different slides were evaluated for each sample. The level of FPG sites was determined using the average value of % DNA in the tail obtained from the sample treated with the enzyme and subtracting the value from the corresponding sample without the enzyme. Four independent experiments were performed.

### Assessment of Histone γ‐H2AX with Flow Cytometry

HBEC cells were exposed to Ni NP, NiO NP and NiCl_2_ for 3 and 24 h at doses of 5, 10, and 25 μg Ni/mL (corresponding to 1.05, 2.1, and 5.26 μg/cm^2^) in 6‐well plates, final volume 2 mL. Cells exposed to 10 μM Camptothecin (Sigma) for 3 h were used as a positive control. The cells were washed with PBS and harvested with trypsin and then fixed in 70% ice cold ethanol and kept at −20°C for at least 1 h, or longer, for fixation. The cells were then spun down and re‐suspended in PBS solution containing 2% FBS. Antibody Anti‐phospho‐Histone H2AX (Ser139) (Merc Millipore, Darmstadt, Germany, clone JBW301, FITC conjugate) was then added to the cells at a final concentration of 1.9 μg/mL and incubated for 1 h at room temperature in the dark. The cells were then washed in PBS solution containing 2% FBS and 10 000 cells/sample was manually recorded with the flow cytometer BD Accuri C6 (BD Biosciences, San Jose, CA, USA). FITC conjugated Anti‐phospho‐Histone H2AX (Ser139) (peak excitation at 494 nm and peak emission at 521 nm) was excited with a 488 laser and analyzed using a 533/30 filter. The BD Accuri C6 software (BD Biosciences) was used for data analysis. Side vs forward scatter was utilized to gate out debris. The positive control was used as a set point to define where cells were considered to be positive for γ‐H2AX. Experiments were repeated three times.

### Cell‐Free and Intracellular ROS Analysis (DCFH‐DA Assay)

The 2′, 7′‐dichloro‐fluorescein diacetate (DCFH‐DA) assay was used to measure cell‐free ROS production as well as total intracellular ROS production. For the cell‐free ROS production, 400 µL NaOH (0.01 M) was added to 10 µL DCFH‐DA (5 mM in DMSO, Sigma‐Aldrich) in an opaque vial for 30 minutes at room temperature to cleave the DA from the DCFH. The reaction was stopped by the addition of 2 mL HBSS (Hank's buffered salt solution). This solution (75 µL) was then mixed with 25 µL NPs/ionic Ni at final concentrations of 10, 25, and 50 μg/mL of Ni and 15 μM DCFH in a black 96‐well plate (Costar, clear bottom). Fluorescence was recorded after 5 min (excitation 485 nm, emission 535 nm) using a plate reader (Tecan Infinite F200, Männedorf, Switzerland, software: Magellan 7.2). ROS generation was calculated as times increase compared to control (HBSS + DCFH).

For the intracellular ROS, 1 × 10^4^ HBEC cells were seeded per well in black and clear bottom 96‐well plates (Corning Incorporated, New York, NY, USA) in triplicates for each exposure, final volume 100 μL. After 24 h, cells were washed with 1× HBSS (Gibco) buffer and incubated with 25 µM DCFH‐DA (Sigma‐Aldrich) for 30 min at 37°C in the dark. The cells were then exposed to Ni, NiO and NiCl_2_ at doses of 5, 10, 25, and 50 μg Ni/mL (corresponding to 1.56, 3.13, 7.81, and 15.63 μg Ni/cm^2^) for 3 h. Cell culture medium was used as negative control and cells exposed to Co NPs (25 μg/mL) were used as a positive control. Fluorescence was recorded every 5 min over 30 min at 37° C with a microplate reader as described above. The average fluorescent intensity for the triplicates was calculated by subtracting the background values. Experiments were repeated three times.

### ToxTracker Reporter Cell Lines Experiments

Six different reporter cell lines; *Srxn1*‐GFP, *Blvrb*‐GFP, *Bscl2*‐GFP, *Rtkn*‐GFP, *Btg2*‐GFP, and *Ddit3*‐GFP were seeded in gelatin‐coated 96‐well plates and exposed to various doses of NPs. The tested NP doses were based on cytotoxicity in mES cells after 24 h exposure to a maximum test dose causing 50–75% cytotoxicity and with a maximum tested dose of 100 μg Ni/mL. After exposure, the particle suspensions were removed and the cells were washed and trypsinized. Induction of the GFP reporters were then measured using a 96‐well Guava flow cytometer (Merck Millipore) as described previously [Hendriks et al., 2012]. Simultaneously, cytotoxicity of the NPs was determined by measuring the concentration of intact cells after exposure using flow cytometry. All presented results show the average GFP induction of at least three independent experiments and error bars represent the standard error of the mean value. The ToxTracker assay is, based on previous validations, considered positive if exceeding a 2 fold increase in the GFP signals [Hendriks et al., 2012].

### *Hprt* Mutation Assay

To test the mutagenic potency of the NPs, the *Hprt* mutation assay was used following exposure of IB10 mES cells as well as V79‐4 cells. The mES cells were exposed to 0.5, 1, 5, and 10 μg Ni/mL (corresponding to 0.21, 1.05, and 2.1 μg/cm^2^) and ENU (*N*‐ethyl‐*N‐*nitrosourea, Sigma‐Aldrich) was used as a positive control at concentrations of 10, 50, and 200 µM. For the V79‐4 cells, Mitomycin C (MMC) was used as a positive control at concentrations of 0.025 and 0.05 μg/mL. Following exposure, 125 000 cells were subcultured in T75 flasks for fixation of mutations during 8 days. The medium was changed every day (mES cells) or every 3–4 days (V79‐4 cells) and the cells were split when confluent. After this, 2–4 million cells (mES) or 1 million cells (V79‐4) were subcultured into 6 wells. At the same time, 100 cells in triplicates were plated for colony forming efficiency (CFE). The cells seeded for mutation scoring were after 3 h treated with 5 μg/mL 6‐thioguanine (Sigma‐Aldrich) to allow the selection of mutated cells. The cells were cultured for 10 days and the medium was changed every 3–4 days with addition of fresh 6‐thioguanine. The colonies formed were then washed twice with PBS and stained with methylene blue (mES cells) or with Giemsa (V79‐4).

Mutation frequency was calculated using the formula:

$$Mutantfrequency(\times 10^{6})=\frac{number\;of\;mutant\;colonies}{Effective\;cell\;number\;seeded}\times 10^{6}$$

where the effective cell number seeded was calculated using the formula:

Effective cell number seeded=Cloning efficiency×cells seeded for mutation

where cloning efficiency was calculated using the formula:

$$Cloning\;efficiency=\frac{No.\;of\;colonies}{\;No.\;of\;cells\;seeded}$$

### Statistical Analysis

Data were analyzed in GraphPad Prism (version 5.02) (La Jolla, CA, USA) by one‐way analysis of variance (ANOVA), with Dunnett's Multiple Comparison Test considering *P*‐values lower than .05 to be statistically significant. The error bars represent the standard error of the mean value.

## RESULTS

### Particle Characterization—Ni Release and Cell‐Free ROS

To complement the previous reported characterization (TEM, surface area and Ni release in serum‐containing DMEM) with data on Ni release in serum‐free medium, ICP‐MS analysis of the Ni release in the HBEC medium (RPMI/LHC‐9) was performed. The results showed on average approx. 2% and 6% (wt%) released Ni in solution following 18 h, from the Ni NPs and the NiO NPs, respectively. The results are compiled in Table 1 together with data from previous studies. The cell‐free ROS measurements showed clear and concentration‐dependent ROS generation from the NiO NPs (5–18 times increase compared to control) whereas the Ni NPs and NiCl_2_ only caused a minor increase in ROS generation (1.1–1.3 times increase from control).

### Size Distribution in Cell Media—PCCS

**Figure 1:**
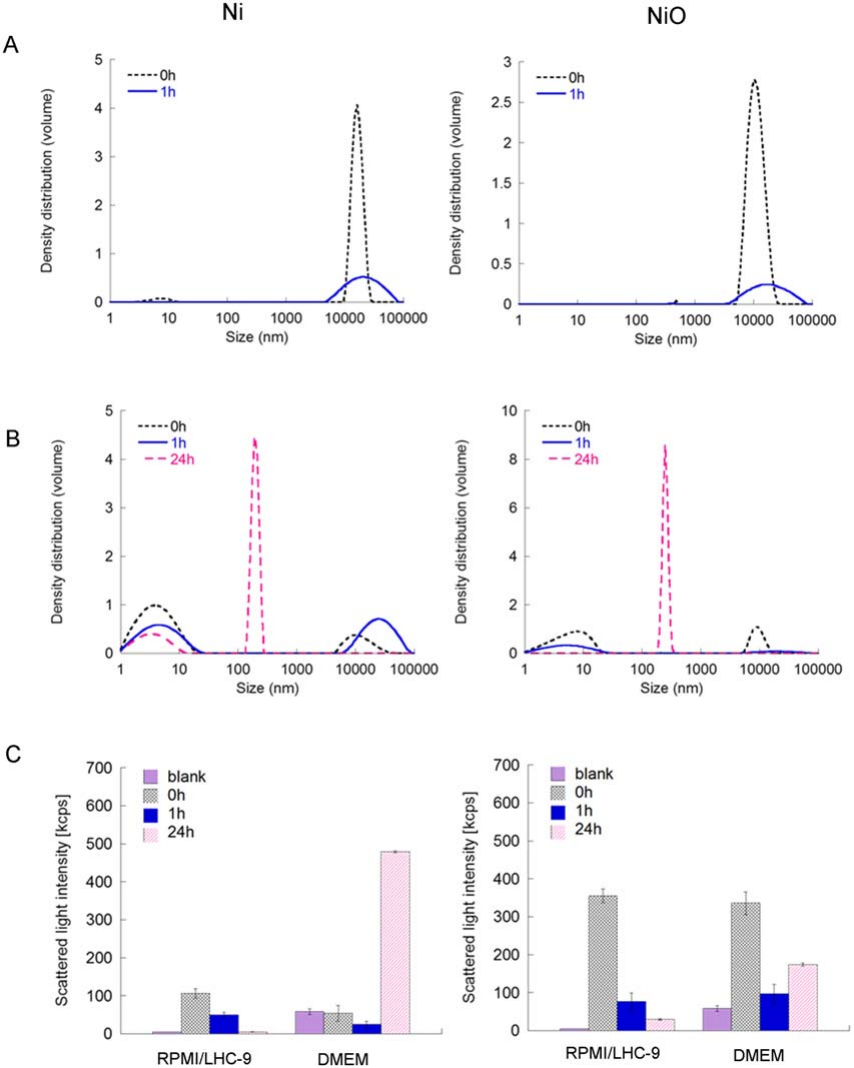
Particle size distributions of Ni and NiO NPs in cell media based on PCCS measurements. (A) size distribution of Ni and NiO NPs in the HBEC medium (LCH‐9/RPMI serum free), (B) size distribution in the reporter cell medium (KnockOut DMEM with serum); (C) intensity of the scattered light in the HBEC medium (left) and reporter cell line medium (right).

Results on the size distribution of the Ni and NiO NPs in serum containing DMEM (KnockOut DMEM used for the ToxTracker reporter cells) as well as in the serum free HBEC medium (RPMI/LHC‐9) are shown in Figure 1 together with corresponding changes in scattered light intensity.

Large agglomerates were formed immediately in the HBEC cell medium for both NPs. After 1 h, the intensities of the scattered light were reduced with only a few large agglomerates still left in the solution. The remaining fraction is believed to have sedimented out from the solution. After 24 h, the scattered light intensities were comparable to that of the blank, and no particles were left in solution, hence no data are shown for this time point.

The size distribution and behavior of the NPs suspended in serum containing DMEM (as used for the reporter cells) were different from observations made in the HBEC medium. For both particle types, a sharp peak appeared at approximately 200 nm after 24 h at the same time as the scattered light intensity increased considerably. This could be related to the release of Ni from the particles that possibly drive proteins in the DMEM to agglomerate and denaturate. This may also explain the reduction in scattered light intensity observed after 1 h (particle sedimentation) and the concomitant strong increase in intensity observed after 24 h (continued Ni release that induced the proteins to agglomerate and scatter more even if no particles were left in solution to scatter the light). Another possibility is that released Ni formed complexes with proteins in the cell media [Latvala et al., 2016]. It is reasonable to assume that several processes take place at the same time, i.e., formation of large complexes/colloids that increase the light intensity and sedimentation of large particles/agglomerates from the solution. The largest agglomerates observed after 0 and 1 h were not present in solution after 24 h.

### Cytotoxicity in HBEC Cells

To test cytotoxic effects of the exposures, the Alamar blue assay was performed at doses 0.5–50 µg/mL of Ni. The results showed that the Ni and NiO NPs were non‐ cytotoxic at the doses tested. NiCl_2_ caused a slight decrease in viability only at the highest dose (Supporting information Fig. S1). Thus, at doses up to 25 µg/mL, all exposures were non‐cytotoxic.

### DNA Strand Breaks following Exposure of HBEC

**Figure 2:**
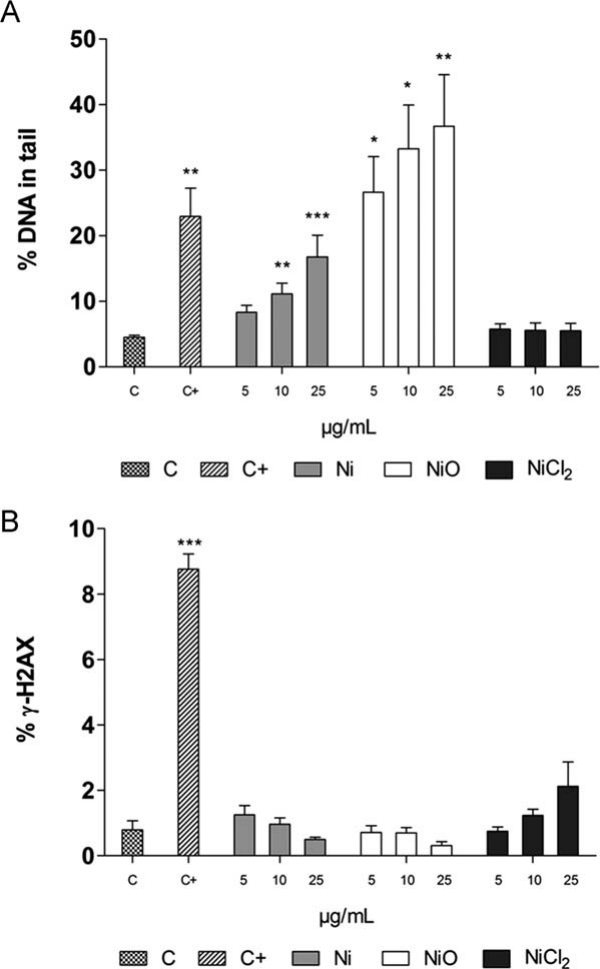
DNA strand breaks following exposure of HBEC cells to Ni NPs, NiO NPs and Ni ions/complexes (from soluble NiCl_2_·H_2_O). The exposures performed for 24 h are reported as µg/mL of Ni. (A) DNA strand breaks shown as % of DNA in tail using the comet assay. Hydrogen peroxide (0.05 M) was used as positive control and showed increased levels in all experiments. (B) DNA double strand breaks measured as % of cells positive for γH2AX staining measured using flow cytometry. Exposure to camptothecin (10 μM) for 3 h was used as a positive control and showed 9% positive cells. The assays were performed in three independent experiments. The bars show mean ± SEM and significant differences compared to the control are marked with asterisks (* for *P*‐value < .05, ** for *P*‐value < .01, *** for *P*‐value < .001).

The level of DNA strand breaks was assessed using comet assay and γ‐H2AX staining. Results are shown in Figure 2. In contrast to the lack of cytotoxicity, the Ni and NiO NPs clearly induced DNA strand breaks, as determined using the comet assay, at the doses tested (5–25 µg/mL), Figure 2A. The NiO NPs were the most potent and caused approx. 26% DNA in the tail already at the lowest dose tested (5 μg/mL of Ni), which was more than 6 times higher than the level in the control cells (4%). Ni NPs caused a significant increase in DNA damage starting at 10 μg/mL (11% DNA in tail). In contrast, no increased level of DNA damage was observed for the added Ni ions (NiCl_2_). A tendency for increased levels of FPG sites was also observed for the Ni and NiO NPs (Supporting information Fig. S2), although the levels were in general quite low and statistically significant only for the Ni NPs at 10 μg/mL. The levels of DNA double strand breaks assessed by using γ‐H2AX staining were assessed using flow cytometry, Figure 2B. None of the Ni exposures caused a significant increase, whereas a clear induction was observed for the positive control (10 μM Camptothecin, 9% positive cells compared to 1% in control). Lack of induction of γ‐H2AX foci was also observed after 3 h exposure (data not shown).

### Intracellular ROS in HBEC

**Figure 3:**
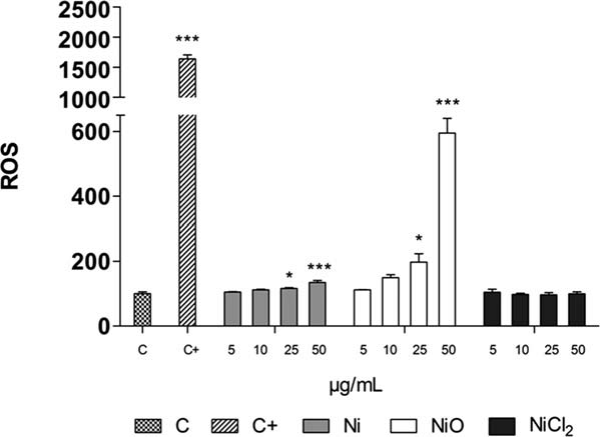
Intracellular ROS in HBEC cells measured using the DCFH‐DA assay. The exposures are reported as µg/mL of Ni and measurements were performed during 30 min after 3 h exposures. Exposure to Co NPs (25 μg/mL) was used as a positive control. Three independent experiments were performed and the bars show mean ± SEM measured as times increase from control. Significant changes, compared to the control, are marked with asterisks (* for *P*‐value < .05, ** for *P*‐value < .01, *** for *P*‐value < .001).

To elucidate whether the increased strand breaks (comet assay) observed could be linked to intracellular ROS generation, the DCFH‐DA assay was performed, Figure 3. A statistically significant increase in intracellular ROS production was observed for the Ni and NiO NPs at higher doses (25 and 50 μg/mL). No significant changes could be observed following the NiCl_2_ exposure.

### ToxTracker Reporter Cell Activation

**Figure 4:**
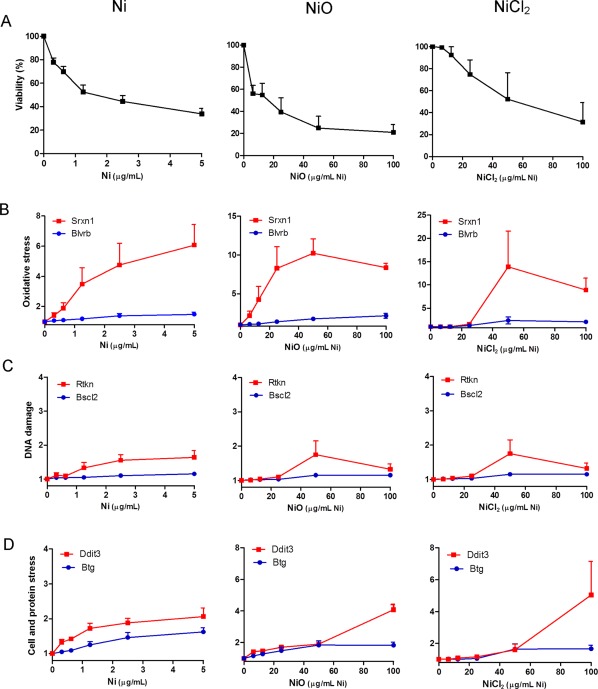
ToxTracker reporter cell line induction for assessing (geno)toxic properties. The reporter cells were exposed to Ni NPs, NiO NPs or Ni ions/complexes (from NiCl_2_·H_2_O) for 24 h and the florescence was measured in intact cells using flow cytometry. The levels shown are fluorescence as times increase compared to control. (A) % viability based on number of intact cells in the *Srxn1* reporter cells, (B) induction of the oxidative stress reporters (*Srxn1* and *Blvrb*), (C) induction of the DNA damage reporters (*Bscl2* and *Rtkn1*), and (D) induction of p53‐related cellular stress (*Btg2*) and protein unfolding (*Ddit3*) reporters. Three independent experiments were performed.

To obtain further mechanistic insight into the (geno)toxicity of the different Ni exposures, we employed six different reporter cell lines (ToxTracker). An initial test of cell viability, measured in terms of fraction of intact cells following 24 h, was first performed in non‐modified mES cells for all exposures up to 100 µg Ni/mL (data not shown). Next, the experiments were performed in the reporter cells with the aim to test up to approx. 50% cytotoxicity. The cell viability showed large differences between Ni NPs, NiO NPs and added Ni ions (NiCl_2_). Most strikingly, the Ni NPs were considerably more toxic than the NiO NPs and the Ni ions, and could therefore only be tested at doses up to 5 μg/mL, whereas the NiO NPs and Ni ions were tested at doses up to 100 μg/mL (of Ni). The cytotoxicity was rather similar among the six different reporter cell lines. Results for the *Srxn1*‐GFP cell line are shown in the viability plot in Figure 4A. All three Ni exposures clearly triggered the oxidative stress reporter *Srxn1*. The induction started already at low doses (below 1 μg/mL) for the Ni NPs, at higher doses (around 5–10 μg/mL) for the NiO NPs and first at 30–50 μg/mL for the Ni‐ions. None of the exposures caused any increase in the *Bscl2* reporter, which suggest that none of NPs or the Ni ions could interact with DNA in a manner that cause stalled replication forks. Only a modest increase in the NFκB signaling reporter (*Rtkn*) was observed. Finally, a clear trend towards induction of protein stress (*Ddit3* reporter) was observed at the highest doses for all three Ni‐containing materials.

### Mutagenic Potential Using *Hprt* Mutation Assay

**Figure 5:**
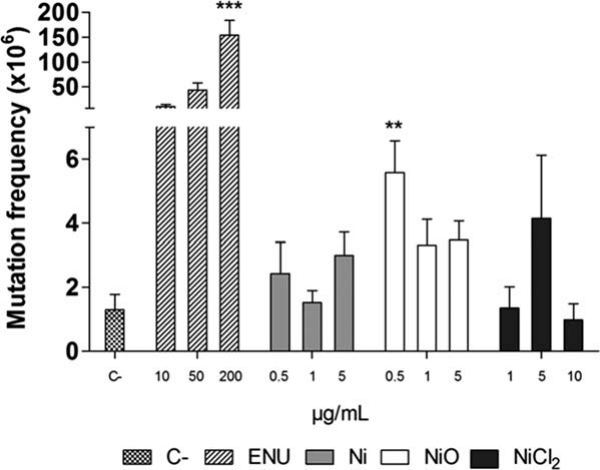
Mutagenic potential of Ni NPs, NiO NPs and Ni ions/complexes (from soluble NiCl_2_·H_2_O) using the *Hprt* mutation assay in mES cells. Three independent experiments were performed and the bars show mean ± SEM. Significant changes, compared to the control, are marked with asterisks (** for *P*‐value < .01, *** for *P*‐value < .001).

The mutagenic potential of the different Ni exposures was examined by the *Hprt* gene mutation assay in mES cells and V79‐4 cells. The mutation frequency per 10^6^ cells (number of mutant colonies per surviving cells) was calculated and the results are presented in Figure 5. The mutation frequency in the mES cells showed a slight increase in the mutant frequency for some of the Ni, NiO and NiCl_2_‐exposures but statistically significant only for one dose (0.5 µg/mL) of NiO. No statistically significan increase in mutation frequency was observed for the V79‐4 cells due to large variation between the experiments (see Supporting information Fig. S3). Since the mutation frequency also varied for the positive control used (MMC), no clear conclusions can be drawn.

## DISCUSSION

The aim of this study was to investigate the genotoxicity of Ni and NiO NPs using a combination of traditional and recently developed methods (ToxTracker reporter cells), and furthermore to compare the effect between the two NPs to Ni‐ions (or Ni‐complexes formed in solution) from soluble NiCl_2_. Our results regarding DNA damage (comet assay) following exposure of HBEC cells show a substantial increase in DNA breaks for the NiO NPs already at the lowest dose of 5 μg/mL, and also a significant increase for the Ni NPs starting at 10 μg/mL. The DNA damage observed was more pronounced compared to that observed in previous studies on A549 cells using the comet assay and the same NPs [Kain et al., 2012; Latvala et al., 2016]. A possible reason could be that A549 cells, which are cancer cells, are rather resistant and insensitive. Another reason could be that the HBEC cells were cultured under serum free conditions, and serum has been shown to protect cells from NP‐induced cytotoxicity and DNA damage [Shi et al., 2012]. Other recent studies have also shown DNA damage using the comet assay following Ni NP exposure of A549 cells (10, 15, and 25 µg/cm^2^) [Magaye et al., 2016], A431 skin cells (2, 4, and 8 μg/mL) [Alarifi et al., 2014] as well as in MCF‐7 breast cancer cells (10, 25, and 50 μg/mL) [Ahamed and Alhadlaq, 2014]. We observed, however, no induction of DNA damage from the easily soluble NiCl_2_ salt (Fig. 2A). One explanation is that the nickel ions will rapidly form complexes with different ligands, e.g., amino acids [Latvala et al., 2016], and such interaction may affect the uptake as well as toxicity. The NPs appeared to mainly cause DNA single strand breaks since no significant increase of double strand breaks was observed when using the γ‐H2AX assay (Fig. 2B). It should be noted, however, that the test was not performed up to cytotoxic doses and effects at higher doses can therefore not be ruled out. A possible limitation of using the flow cytometric γ‐H2AX assay is that particles may shield the fluorescence from the γ‐H2AX antibody. This cannot be totally ruled out, but fluorescence was clearly measured using flow cytometry when employing the reporter cells, suggesting no large effect of shielding. DNA single strand breaks may be formed as a result of intracellular ROS, and in line with this hypothesis, we found increased ROS levels after exposure to the higher doses of the Ni and NiO NPs (Fig. 3). The more pronounced effect of NiO at 50 µg/mL is likely explained by an inherent ability of these NPs to generate ROS, as noted from the cell‐free measurements. In contrast, we could only detect a small, non‐significant increase in oxidatively damaged DNA (FPG sites) for the NiO NPs and a significant increase at one dose of the Ni NPs. The low induction of FPG sites for these NiO NPs is in line with our previous studies on the same batch of NPs [Kain et al., 2012; Karlsson et al., 2014]. Clearly, a higher induction at higher doses is possible.

Oxidative stress as an important underlying (geno)toxic mechanism is also supported by the ToxTracker reporter cell lines. These reporter cells have been extensively validated using the compound library suggested by the European Centre for the Validation of Alternative Methods (ECVAM) [Hendriks et al., 2016]. This validation showed, for example, that almost all genotoxic carcinogens (ECVAM class 1) induced the DNA damage reporters. These reporters do not identify DNA single strand breaks and repair intermediates, damage that is measured by the comet assay, but the *Rtkn* reporter will be activated by DNA double strand breaks. Thus, agents that predominantly act via oxidative stress causing DNA single strand breaks will mainly activate the oxidative stress reporters. The Ni and NiO NPs triggered mainly the induction of the oxidative stress reporter (Fig. 4). These observations are in line with our previous study on NiO and CuO NPs using three of the reporters [Karlsson et al., 2014].

It was also striking that the mES cells were very sensitive to metal Ni NPs since clear effects (on viability and oxidative stress) were observed at 5–10 times lower doses than NiO NPs and Ni ions/complexes. In general, effects from Ni ions were mainly observed at high doses. The uptake of the Ni ions is possibly limited at low (non‐cytotoxic) doses but at higher doses the membrane integrity may be affected, allowing penetration of Ni into the cells causing oxidative stress as well as protein stress (*Ddit3* reporter) as observed for the highest dose (Fig. 4). Another explanation may be related to the formation of Ni complexes/colloids in solution that act as NPs with facilitated uptake via endocytotic mechanisms at the highest Ni doses. Protein stress was also observed for both Ni and NiO NPs at the highest doses. Such an effect was also suggested in a recent study using a proteomic approach following exposure of BEAS‐2B cells to Ni(II) [Ge et al., 2016]. The study showed an upregulation of the ubiquitin protein C (UBC), a protein involved in the ubiquitin‐proteasome pathways that catalyze the rapid elimination of proteins with abnormal conformation [Ge et al., 2016]. Besides this effect, the same study also highlighted changes in key proteins involved in glycolysis and gluconeogenesis pathways, apoptosis and stress responses including inflammation and oxidative stress [Ge et al., 2016].

Finally, to test whether the Ni and NiO NPs could cause mutations, we employed the OECD accepted *Hprt* mutation assay following exposure of mES cells. Indeed, since the use of bacterial assays such as Ames test most likely can underestimate mutagenic effects due to lack of uptake [Doak et al., 2012], future studies should employ assays using mammalian cells. In general, however, the number of studies using the *Hprt* assay for testing NPs is still limited. The assay was recently used for testing different synthetic amorphous silica nanomaterials showing no mutagenic activity [Guichard et al., 2016], whereas gene mutations were, for example, observed for Ag NPs [Huk et al., 2015]. We observed a statistically significant increase in mutations for NiO but only for one of the doses (0.5 µg/mL), see Figure 5. The assay was also performed following exposure of V79‐4 and the results suggested a weak increase in mutations, but due to high variation and low response in the positive control used (MMC), the data were not further considered (Supporting information Fig. S3). In general, Ni is considered to be a weakly mutagenic agent [IARC, 2012], and the results generated in our study are thus in agreement with this statement. A previous study showed that black NiO (not NPs), i.e., NiO of non‐stoichimetric composition, and NiCl_2_ did not induce any mutagenic response in V79‐4‐cells, but the transgenic cell line G12 responded very strongly to the rather insoluble Ni compounds (NiS, Ni_3_S_2_, and NiO) in the *Hprt* mutation assay [Kargacin et al., 1993]. Furthermore, NiS, Ni_3_S_2_, and NiO were also shown to strongly induce mutations in the G10 transgenic cell line, where also NiCl_2_ was mutagenic, although to a lesser degree [Klein et al., 1994].

Taken together, this study showed DNA damage, mainly in the form of DNA single strand breaks, in lung epithelial cells (cultured at serum free conditions) to be induced by exposure to Ni and NiO NPs, whereas no effect was observed for Ni ions/complexes introduced as soluble NiCl_2_. Furthermore, a small but statistically significant increase in *Hprt* mutations was observed for NiO but only at one dose. By employing six different reporter cell lines, oxidative stress was concluded to be the main (geno)toxic mechanism and protein unfolding also occurred at higher doses. None of the Ni‐containing materials induced the DNA damage reporter related to direct DNA damage and stalled replication forks. The results are summarized in Table 2. We conclude that the Ni and NiO NPs show more pronounced (geno)toxic effects compared to Ni ions/complexes indicating more serious health concerns. Identification of oxidative stress as primary mechanism of genotoxicity suggests lower carcinogenic hazard and a threshold dose‐response compared to agents that interact directly with DNA.

**Table 2:** Concluding Table of Results

	Ni	NiO	NiCl_2_
Cytotoxicity HBEC ≤50 µg/mL	No	No	Minor at 50 µg/mL
DNA strand breaks HBEC ≤25 µg/mL	Yes from 10 µg/mL	Yes from 5 µg/mL	No
DNA double strand breaks HBEC ≤25 µg/mL	No	No	No
Cell‐free ROS	Minor	Yes	Minor
Intracellular ROS HBEC ≤50 µg/mL	Minor	Yes	No
Cytotoxicity reporter cells	Yes	Yes	Yes
Oxidative stress reporter induction	Yes	Yes	Yes
DNA damage reporter induction	No^a^	No^a^	No^a^
Protein stress reporter induction	Yes, at high cytotoxicity	Yes, at high cytotoxicity	Yes at high cytotoxicity
*Hprt* mutations mES cells ≤5 µg/mL	No	At one dose	No

a A modest increase in *Rtkn* reporter but not reaching the ×2 threshold.

## AUTHOR CONTRIBUTIONS

EÅ was involved in the study design, performed the comet assay experiments, the γH2AX experiments, the *Hprt* mutation assay and CFE experiments (V79‐4 cells), prepared samples for PCCS analysis, performed statistical analysis and drafted the manuscript. FC performed all ToxTracker reporter cell experiments, SDB designed and performed the *Hprt* mutation assay (V79‐4 cells), SI performed the Alamar blue and DCFH‐DA assays as well as ICP‐analysis of Ni release, SS performed the PCCS analysis, RD performed the *Hprt* mutation assay (mES cells), IOW supervised the particle characterization, GH supervised all experiments and interpretations related to the ToxTracker reporter cell lines, HLK initiated the study, performed the cell‐free ROS measurements, had the main responsibility for study design, interpretations and for finalizing the manuscript. All authors critically reviewed the manuscript and approved the final result interpretation.

## CONFLICT OF INTEREST

GH and RD are employed by Toxys, a Dutch company that offers ToxTracker as a commercial service.

## Supporting information

Supporting Information

## References

[em22163-bib-0001] Ahamed M , Alhadlaq HA. 2014 Nickel nanoparticle‐induced dose‐dependent cyto‐genotoxicity in human breast carcinoma MCF‐7 cells. Oncotargets Ther 7:269–280. 10.2147/OTT.S58044 PMC3931666 24627639

[em22163-bib-0002] Alarifi S , Ali D , Alakhtani S , Al Suhaibani ES , Al‐Qahtani AA. 2014 Reactive oxygen species‐mediated DNA damage and apoptosis in human skin epidermal cells after exposure to nickel nanoparticles. Biol Trace Elem Res 157:84–93. 24307203 10.1007/s12011-013-9871-9

[em22163-bib-0003] Costa M , Davidson TL , Chen H , Ke Q , Zhang P , Yan Y , Huang C , Kluz T. 2005 Nickel carcinogenesis: epigenetics and hypoxia signaling. Mutat Res 592:79–88. 16009382 10.1016/j.mrfmmm.2005.06.008

[em22163-bib-0004] Di Bucchianico S , Cappellini F , Le Bihanic F , Zhang YN , Dreij K , Karlsson HL. 2017 Genotoxicity of TiO_2_ nanoparticles assessed by mini‐gel comet assay and micronucleus scoring with flow cytometry. Mutagenesis 32:127–137. 27382040 10.1093/mutage/gew030 PMC5180169

[em22163-bib-0005] Doak SH , Manshian B , Jenkins GJS , Singh N. 2012 In vitro genotoxicity testing strategy for nanomaterials and the adaptation of current OECD guidelines. Mutat Res Gen Toxicol Environ 745:104–111. 10.1016/j.mrgentox.2011.09.013 PMC4028084 21971291

[em22163-bib-0006] Ge Y , Bruno M , Haykal‐Coates N , Wallace K , Andrews D , Swank A , Winnik W , Ross JA. 2016 Proteomic assessment of biochemical pathways that are critical to nickel‐induced toxicity responses in human epithelial cells. PloS One 11:e0162522. 27626938 10.1371/journal.pone.0162522 PMC5023113

[em22163-bib-0007] Giachino C , Orlando L , Turinetto V. 2013 Maintenance of genomic stability in mouse embryonic stem cells: relevance in aging and disease. Int. J. Mol. Sci 14:2617–2636. 23358251 10.3390/ijms14022617 PMC3588006

[em22163-bib-0008] Goodman JE , Prueitt RL , Thakali S , Oller AR. 2011 The nickel ion bioavailability model of the carcinogenic potential of nickel‐containing substances in the lung. Crit Rev Toxicol 41:142–174. 21158697 10.3109/10408444.2010.531460

[em22163-bib-0009] Guichard Y , Fontana C , Chavinier E , Terzetti F , Gate L , Binet S , Darne C. 2016 Cytotoxic and genotoxic evaluation of different synthetic amorphous silica nanomaterials in the V79 cell line. Toxicol Ind Health 32:1639–1650. 25757481 10.1177/0748233715572562

[em22163-bib-0010] Hendriks G , Atallah M , Morolli B , Calleja F , Ras‐Verloop N , Huijskens I , Raamsman M , van de Water B , Vrieling H. 2012 The ToxTracker assay: novel GFP reporter systems that provide mechanistic insight into the genotoxic properties of chemicals. Toxicol Sci 125:285–298. 22003191 10.1093/toxsci/kfr281

[em22163-bib-0011] Hendriks G , Derr RS , Misovic B , Morolli B , Calleja FMGR , Vrieling H. 2016 The extended ToxTracker assay discriminates between induction of DNA damage, oxidative stress, and protein misfolding. Toxicol Sci 150:190–203. 26719371 10.1093/toxsci/kfv323 PMC5009621

[em22163-bib-0012] Huk A , Izak‐Nau E , el Yamani N , Uggerud H , Vadset M , Zasonska B , Duschl A , Dusinska M. 2015 Impact of nanosilver on various DNA lesions and HPRT gene mutations ‐ effects of charge and surface coating. Part Fibre Toxicol 12:25. 26204901 10.1186/s12989-015-0100-x PMC4513976

[em22163-bib-0013] IARC . 1990. IARC monographs on the evaluation of carcinogenic risks to humans. 10.1289/ehp.94102590 PMC1569767 9679121

[em22163-bib-0014] IARC . 2012. IARC monographs on nickel and nickel compounds. Volume 100C.

[em22163-bib-0015] Kain J , Karlsson HL , Moller L. 2012 DNA damage induced by micro‐ and nanoparticles–interaction with FPG influences the detection of DNA oxidation in the comet assay. Mutagenesis 27:491–500. 22447192 10.1093/mutage/ges010

[em22163-bib-0016] Kargacin B , Klein CB , Costa M. 1993 Mutagenic responses of nickel oxides and nickel sulfides in Chinese hamster V79 cell lines at the xanthine‐guanine phosphoribosyl transferase locus. Mutat Res 300:63–72. 7683771 10.1016/0165-1218(93)90141-y

[em22163-bib-0017] Karlsson HL , Gliga AR , Calleja FM , Goncalves CS , Wallinder IO , Vrieling H , Fadeel B , Hendriks G. 2014 Mechanism‐based genotoxicity screening of metal oxide nanoparticles using the ToxTracker panel of reporter cell lines. Part Fibre Toxicol 11:41. 25179117 10.1186/s12989-014-0041-9 PMC4237954

[em22163-bib-0018] Kawanishi S , Inoue S , Oikawa S , Yamashita N , Toyokuni S , Kawanishi M , Nishino K. 2001 Oxidative DNA damage in cultured cells and rat lungs by carcinogenic nickel compounds. Free Radical Bio Med 31:108–116. 11425496 10.1016/s0891-5849(01)00558-5

[em22163-bib-0019] Klein CB , Kargacin B , Su L , Cosentino S , Snow ET , Costa M. 1994 Metal mutagenesis in transgenic Chinese hamster cell lines. Environ Health Perspect 102: 63–67. 10.1289/ehp.94102s363 PMC1567392 7843139

[em22163-bib-0020] Latvala S , Hedberg J , Di Bucchianico S , Moller L , Odnevall Wallinder I , Elihn K , Karlsson HL. 2016 Nickel Release, ROS Generation and Toxicity of Ni and NiO Micro‐ and Nanoparticles. PloS One 11:e0159684. 27434640 10.1371/journal.pone.0159684 PMC4951072

[em22163-bib-0021] M'Bemba‐Meka P , Lemieux N , Chakrabarti SK. 2005 Nickel compound‐induced DNA single‐strand breaks in chromosomal and nuclear chromatin in human blood lymphocytes in vitro: Role of oxidative stress and intracellular calcium. Mutat Res Gen Toxicol Environ 586:124–137. 10.1016/j.mrgentox.2005.06.001 16099703

[em22163-bib-0022] Magaye R , Gu Y , Wang Y , Su H , Zhou Q , Mao G , Shi H , Yue X , Zou B , Xu J , and others. 2016 In vitro and in vivo evaluation of the toxicities induced by metallic nickel nano and fine particles. J Mol Histol 47:273–286. 27010930 10.1007/s10735-016-9671-6

[em22163-bib-0023] Magaye R , Zhao JS. 2012 Recent progress in studies of metallic nickel and nickel‐based nanoparticles' genotoxicity and carcinogenicity. Environ Toxicol Phar 34:644–650. 10.1016/j.etap.2012.08.012 23000472

[em22163-bib-0024] Nelson BC , Wright CW , Ibuki Y , Moreno‐Villanueva M , Karlsson HL , Hendriks G , Sims CM , Singh N , Doak SH. 2016 Emerging metrology for high‐throughput nanomaterial genotoxicology. Mutagenesis 32:215–232. 27565834 10.1093/mutage/gew037 PMC5500194

[em22163-bib-0025] Oller AR , Oberdorster G , Seilkop SK. 2014 Derivation of PM10 size‐selected human equivalent concentrations of inhaled nickel based on cancer and non‐cancer effects on the respiratory tract. Inhal Toxicol 26:559–578. 25055843 10.3109/08958378.2014.932034

[em22163-bib-0026] Ramirez RD , Sheridan S , Girard L , Sato M , Kim Y , Pollack J , Peyton M , Zou Y , Kurie JM , DiMaio JM , and others. 2004 Immortalization of human bronchial epithelial cells in the absence of viral oncoproteins. Cancer Res 64:9027–9034. 15604268 10.1158/0008-5472.CAN-04-3703

[em22163-bib-0027] Schram SE , Warshaw EM , Laumann A. 2010 Nickel hypersensitivity: a clinical review and call to action. Int J Dermatol 49:115–125. 20465634 10.1111/j.1365-4632.2009.04307.x

[em22163-bib-0028] Schwerdtle T , Seidel A , Hartwig A. 2002 Effect of soluble and particulate nickel compounds on the formation and repair of stable benzo[a]pyrene DNA adducts in human lung cells. Carcinogenesis 23:47–53. 11756222 10.1093/carcin/23.1.47

[em22163-bib-0029] Shi J , Karlsson HL , Johansson K , Gogvadze V , Xiao L , Li J , Burks T , Garcia‐Bennett A , Uheida A , Muhammed M , et al. 2012 Microsomal glutathione transferase 1 protects against toxicity induced by silica nanoparticles but not by zinc oxide nanoparticles. ACS Nano 6:1925–1938. 22303956 10.1021/nn2021056 PMC3314313

