# Supplementary material for: Genotoxic and mutagenic properties of Ni and NiO nanoparticles investigated by comet assay, γ‐H2AX staining, Hprt mutation assay and ToxTracker reporter cell lines
Source: Environ Mol Mutagen. 2017 Dec 15;59(3):211–22. doi: 10.1002/em.22163 (PMC5888189; doi:10.1002/em.22163)
Supplement: Supplementary file 1 — Supporting Information [file EM-59-211-s001.docx]

**Supporting information**

**Genotoxic and mutagenic properties of Ni and NiO nanoparticles investigated in three different *in vitro* model systems**

Emma Åkerlund^1^, Francesca Cappellini^1^, Sebastiano Di Bucchianico^1^, Md Shafiqul Islam^1^, Sara Skoglund^2^, Remco Derr^3^, Inger Odnevall Wallinder^2^, Giel Hendriks^3^ and Hanna L. Karlsson^1*^

^1^Unit of Biochemical Toxicology, Institute of Environmental Medicine, Karolinska Institutet, 171 77 Stockholm, Sweden

^2^KTH Royal Institute of Technology, Division of Surface and Corrosion Science, School of Chemical Science and Engineering, Stockholm, Sweden

^3^Toxys, Robert Boyleweg 4, 2333 CG Leiden, the Netherlands

**Suppl. Fig. 1.**


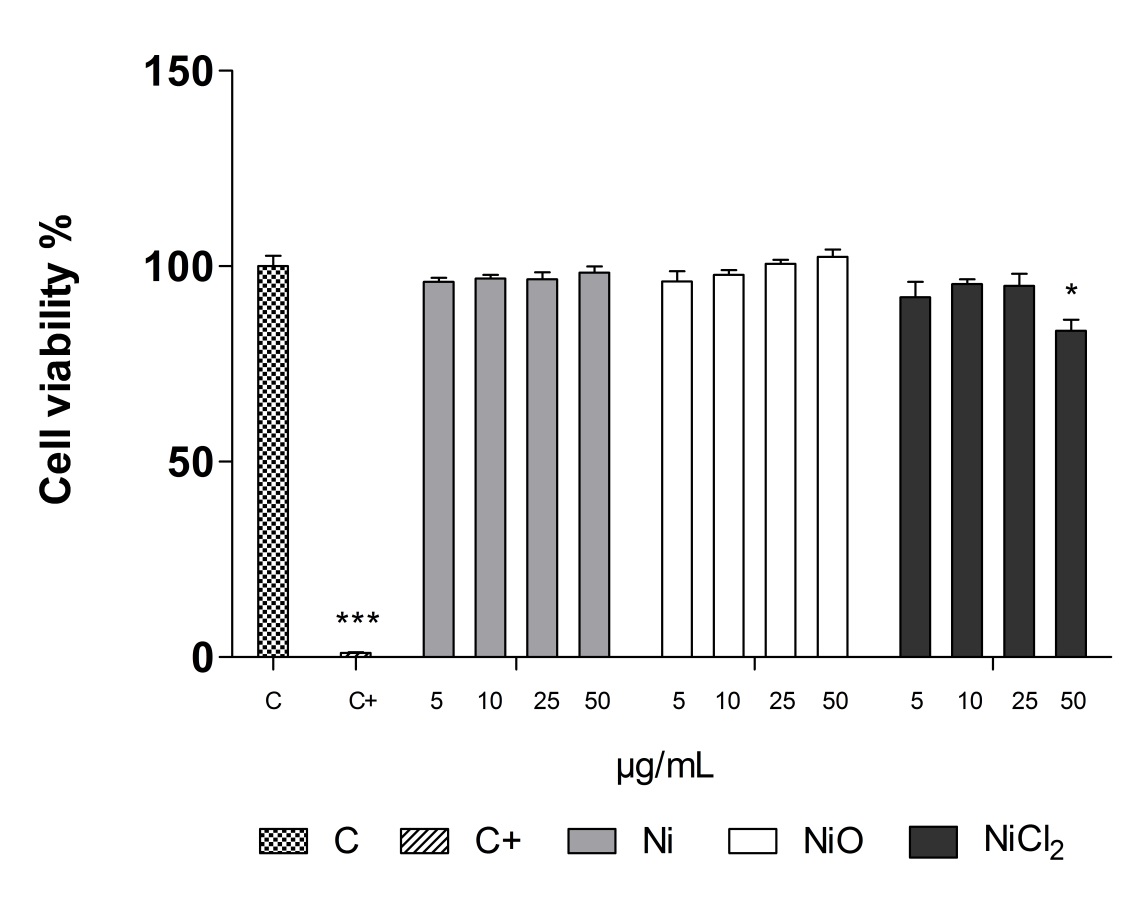


**Suppl. Fig. 1.** Cell viability of HBEC cells measured using the Alamar blue assay following exposure of Ni NPs, NiO NPs and NiCl_2_ for 24 h. Three independent experiments were performed and the bars show mean ± SEM. Significant changes, compared to the control, are marked with asterisks (* for p-value < 0.05, *** for p-value < 0.001).

**Suppl. Fig. 2.**


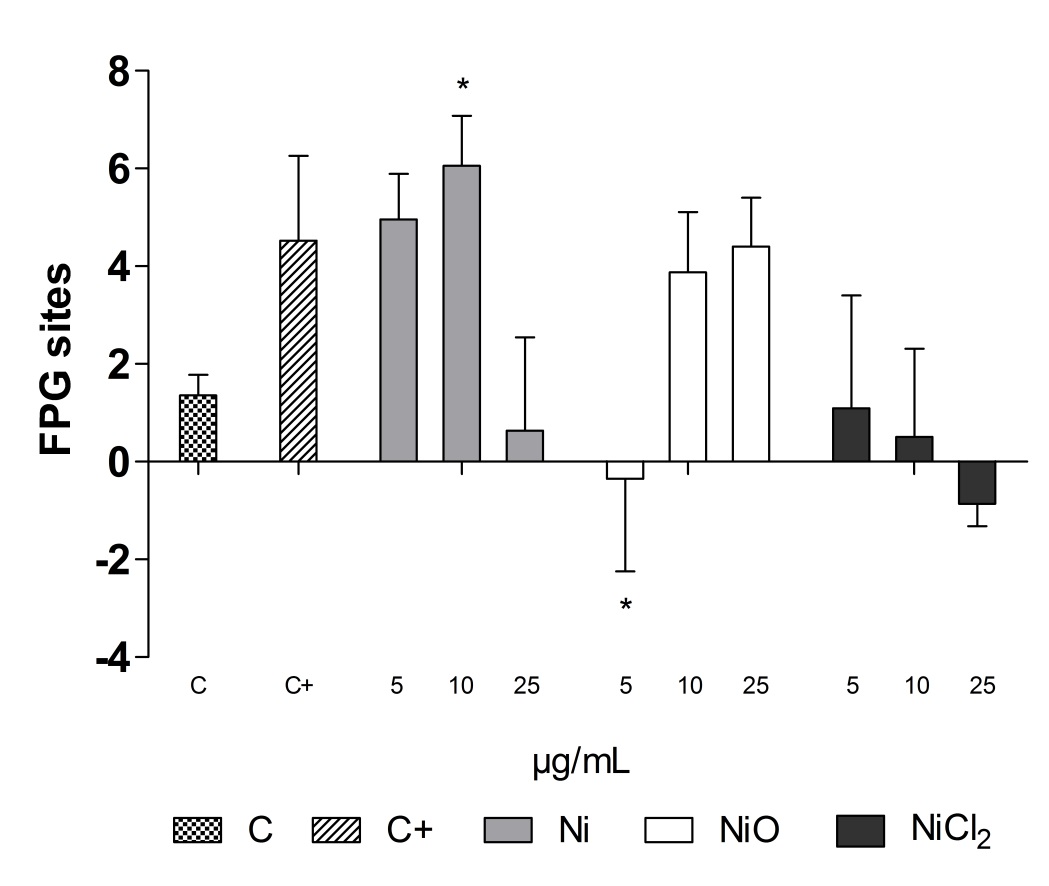


**Suppl. Fig. 2.** FPG sites following exposure of HBEC cells to Ni NPs, NiO NPs and NiCl_2_. The exposures were performed for 24 h and are reported as µg/mL of Ni. Three independent experiments were performed and the bars show mean ± SEM. Significant changes, compared to the control, are marked with asterisks (* for p-value < 0.05).

**Suppl. fig. 3.**


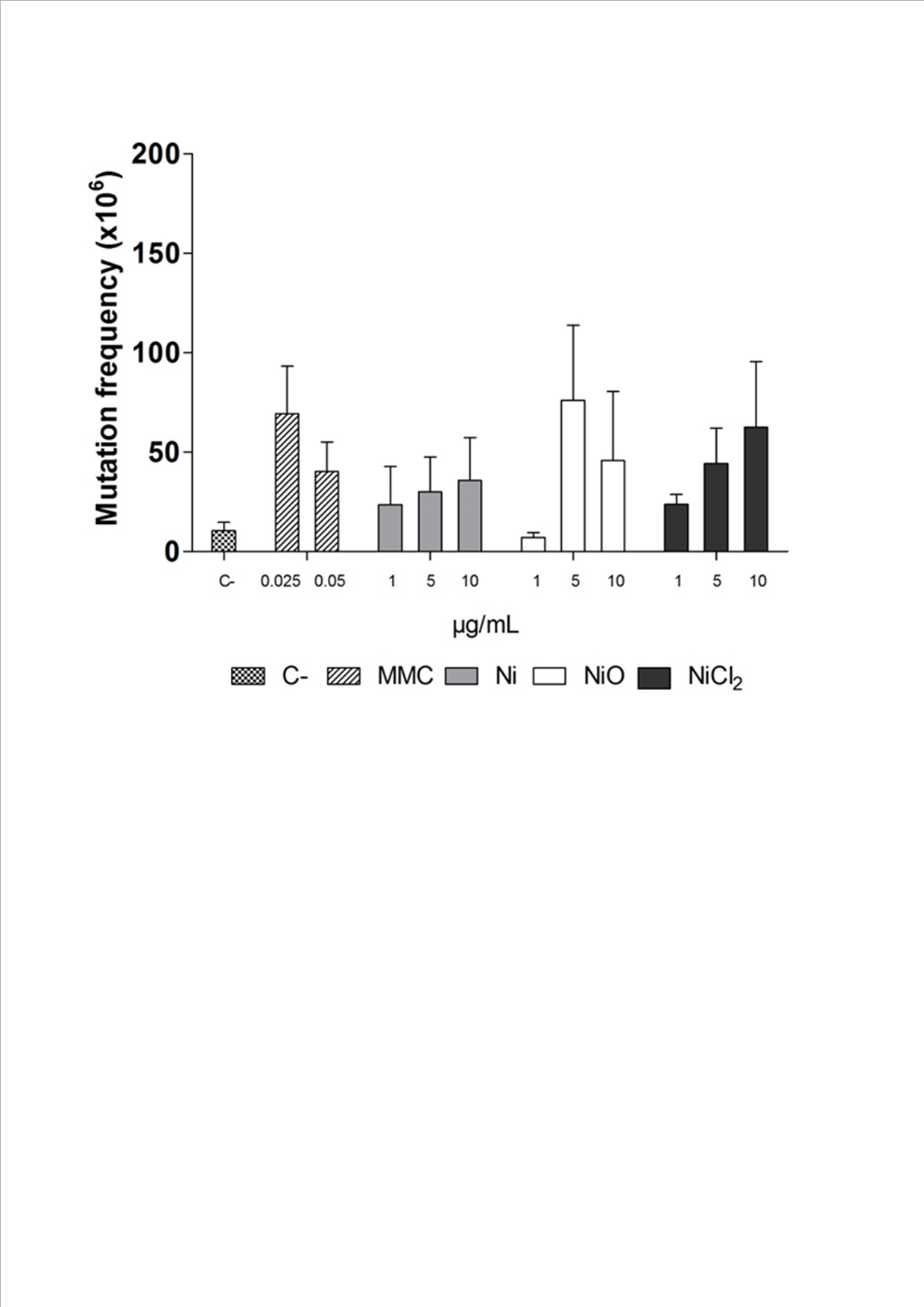


**Suppl. Fig. 3.** Mutagenic potential of Ni NPs, NiO NPs and Ni ions/complexes (from soluble NiCl2·H2O) using the *Hprt* mutation assay following exposure of V79-4 cells. Three independent experiments were performed and the bars show mean ± SEM.
